# A Profile of Black and Latinx Older Adults Receiving Care in Nursing Homes: 2011-2017

**DOI:** 10.1093/geroni/igab046.923

**Published:** 2021-12-17

**Authors:** Jasmine Travers, Andrew Dick, Bei Wu, David Grabowski, Mansi Agarwal, Gayani Perera, Patricia Stone

**Affiliations:** 1 New York University, New York, New York, United States; 2 Rand Corporation, Boston, Massachusetts, United States; 3 Harvard Medicaid School, Boston, Massachusetts, United States; 4 Division of Biostatistics, Washington University School of Medicine, St Louis, Missouri, United States; 5 Center for Health Policy Columbia University School of Nursing, New York, New York, United States; 6 Columbia University School of Nursing, New York, New York, United States

## Abstract

Between the years 1999-2008, a substantial increase in nursing home use occurred among Black and Latinx older adults, while white older adults’ use of nursing homes decreased. These disparate trends suggested potential racial and ethnic disparities in options for preferred long-term services and supports (LTSS) settings. Over the last decade, several initiatives have been put in place to support LTSS needs in the community. However, it is unclear whether Black and Latinx older adults are continuing to use nursing home services at disproportionate rates. We used LTCfocus data for 2011-2017 to explore current trends in nursing home use and access among Black and Latinx older adults in light of these current initiatives. Our findings reveal a continued rise in Black and Latinx older adults’ use of nursing homes while white older adults’ use continues to decline. More notably, there has been a decline in nursing homes servicing these minority groups.

